# Morphological and Morphometric Assessment of Adolescent Idiopathic Scoliosis According to Pelvic Axial Rotation—A Retrospective Cohort Study with 397 Patients

**DOI:** 10.3390/children12080991

**Published:** 2025-07-28

**Authors:** Nevzat Gönder, Cansu Öztürk, Rabia Taşdemir, Zeynep Şencan, Cağrı Karabulut, Ömer Faruk Cihan, Musa Alperen Bilgin

**Affiliations:** 1 Department of Orthopedics and Traumatology, Faculty of Medicine, Gaziantep University, Gaziantep 27000, Turkey; 2 Department of Anatomy, Faculty of Medicine, Gaziantep Islam Science and Technology University, Gaziantep 27000, Turkey; cansu.ozturk@gibtu.edu.tr (C.Ö.); rabia.tasdemir@gibtu.edu.tr (R.T.); 3 Department of Anatomy, Faculty of Medicine, Gaziantep University, Gaziantep 27000, Turkey; zeynepsencan@gantep.edu.tr (Z.Ş.); ofcihan@gantep.edu.tr (Ö.F.C.); 4 Department of Orthopaedics and Traumatology, T.C. Ministry of Health Pazarcık State Hospital, Kahramanmaraş 46700, Turkey; cagri.karabulut@saglik.gov.tr; 5 Department of Orthopedics and Traumatology, Çorum Iskilip State Hospital, Çorum 19400, Turkey; musaalperen.bilgin@saglik.gov.tr

**Keywords:** adolescent idiopathic scoliosis, complex spine, pelvic axial rotation, morphometry, cobb angle, risser classification, lenke classification

## Abstract

**Background:** A precise radiographic evaluation of adolescent idiopathic scoliosis (AIS) is essential for effective treatment planning and follow-up. The pelvic axial rotation (PAR) and horizontal balance of the pelvis are critical factors to consider throughout the treatment and monitoring of AIS. While some previous studies have examined spinal curvature in relation to PAR direction and the direction of the major curve (DMC) in AIS patients, this study aims to explore the relationship between scoliosis morphology, pelvic axial rotation (PAR), and the direction of the major curve in patients with adolescent idiopathic scoliosis. **Methods:** Radiographic images of 397 patients diagnosed with AIS between 2023 and 2024 at a Tertiary Referral Hospital were retrospectively evaluated. Morphological and morphometric measurements, including sex, Lenke and Risser classifications, lower leg discrepancy, Cobb angle, PAR direction, and major curvature direction, were performed. **Results:** The mean age of the 397 patients (246 female, 151 male) was 14.47 ± 2.29. There is no significant correlation between PAR and DMC (*p* = 0.919). No significant differences were found in terms of sex (*p* = 0.603). Regardless of the PAR direction, major curvature was more common on the left side (57.7%). Furthermore, a positive correlation was noted between the Cobb angle and LLD. **Conclusions:** Our study contributes to a growing body of literature questioning the deterministic role of PAR in AIS. While previous reports have emphasized the directional correlation between the pelvis and spinal curvature, our findings suggest that pelvic rotation may not be a reliable indicator of curve direction in all patients. This highlights the complexity of AIS biomechanics and underscores the need for individualized radiographic and clinical evaluation rather than a reliance on generalized compensatory models.

## 1. Introduction

The vertebral column plays a critical structural and functional role in the human body, supporting the organs of the thoracic, abdominal, and cranial cavities. Alongside this, the pelvis forms the biomechanical foundation of the body, serving as the interface between the axial skeleton and the lower extremities [1]. In an upright position, the pelvis transmits the body’s weight from the spine to the lower limbs, while in a seated position, the ischial tuberosities assume this function. It is also the anchoring point for numerous muscles of the lower extremities and contributes significantly to postural balance and stability [1]. Given these essential roles, the pelvis possesses a structurally robust design. However, any morphological alteration or functional insufficiency in the pelvis can disrupt its relationship with the lumbar spine and lower limbs, potentially compromising postural alignment and dynamic balance [1,2].

Scoliosis is a complex three-dimensional spinal deformity, defined by a lateral deviation of the vertebral column greater than 10° in the coronal plane, with concurrent alterations in all three planes [3]. The idiopathic form, first described by Kleinberg in 1922, is diagnosed when no identifiable cause of the spinal curvature can be found [3]. Among idiopathic cases, adolescent idiopathic scoliosis (AIS) is the most common subtype, with a global prevalence that varies significantly by region—reported as 3.9% in North America, 1.9%, 0.1% in Israel, 7.5% in Spain, and 1.8% in Turkey [4,5,6,7]. Ethnic variations in AIS incidence, disease severity, and patient outcomes—including satisfaction and quality of life after treatment—have been reported [4,5,6,7].

Radiographic studies have shown that pelvic rotation often occurs toward the side of the major thoracic curvature in AIS patients [8]. Furthermore, asymmetrical pelvic positioning has been linked to alterations in femoral morphology, such as an increased neck-shaft angle on the contralateral side [2]. Although it is well established that scoliosis can induce pelvic rotation, relatively few studies have comprehensively examined how spinal curvature morphology is affected in relation to the direction of pelvic axial rotation (PAR). Understanding this relationship is critical for treatment planning, as PAR can influence both spinal alignment and lower limb biomechanics.

Pelvic axial rotation (PAR) in AIS has garnered increasing attention due to its presumed role in three-dimensional spinal balance and compensatory mechanisms. Seminal works such as those by Gum et al. (2007), Wang et al. (2014), and Zuckerman et al. (2021) reported a frequent concordance between the direction of thoracic curve rotation and the direction of pelvic rotation [1,8,9]. These studies posited that the pelvis rotates to the same direction as the major curve either due to a compensatory mechanism or intrinsic pathoanatomic torsion, with many interpreting PAR as a reflection of transverse compensation for spinal deformity. Authors Asher et al. (2010) and Zhao et al. (2016) propose an opposite viewpoint [10,11].

We hypothesized that the direction of PAR would correlate with the direction of the major scoliotic curve (DMC) and that the leg length discrepancy (LLD) would be associated with PAR and the Lenke classification. Our objective is to offer insights that may facilitate more personalized care approaches and make significant additions to the existing body of literature by examining the interplay between scoliosis morphology, PAR direction, and major curve orientation.

## 2. Materials and Methods

### 2.1. Study Design and Setting

**Figure 1:**
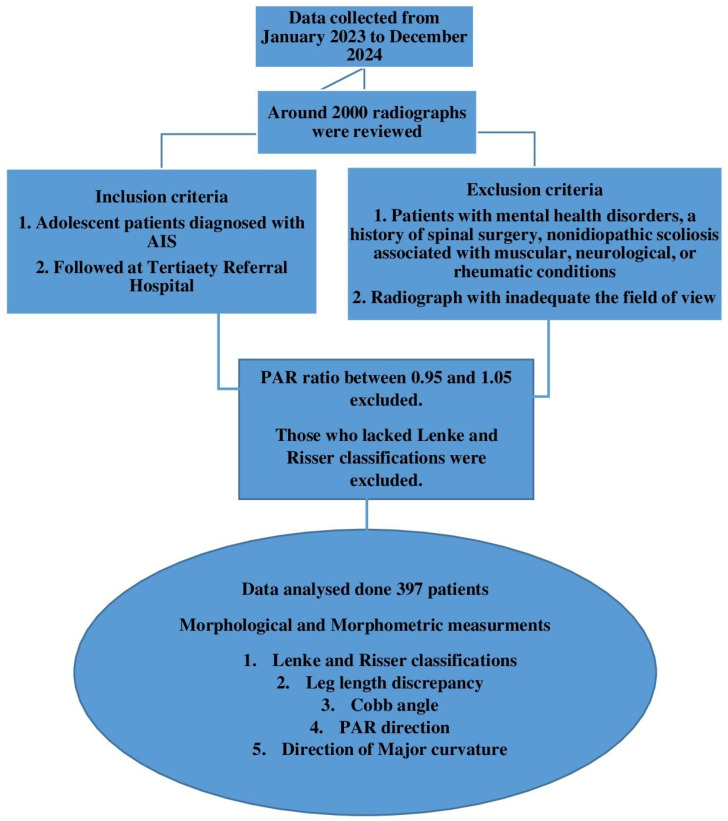
Flowchart illustrating the sample selection.

This retrospective, cross-sectional study was conducted using archived radiographic images of patients diagnosed with adolescent idiopathic scoliosis (AIS) who received treatment at a Tertiary Referral Hospital between January 2023 and December 2024. The study was conducted in accordance with the Declaration of Helsinki. Ethical approval dated 15 May 2024 and numbered 2024/162 was obtained from the Ethics Committee for Non-Invasive Clinical Research of Gaziantep University. Approximately 2000 radiographs were reviewed during the archive search. The study was conducted following STROBE guidelines for retrospective cross-sectional studies [12] (Figure 1).

### 2.2. Study Population

The sample size was calculated using Cochran’s formula for large populations, with a 95% confidence level (Z = 1.96), an estimated proportion of 0.5, and a 5% margin of error (E = 0.05). The final sample size needed for this study was 384 [13,14].

A total of 397 adolescent patients who were diagnosed with AIS and had received treatment at the Orthopedics and Traumatology Clinic of a Tertiary Referral Hospital were included in the study. The mean age of the patients was 14.47 ± 2.29 years. Any images in the archive that satisfied this criteria were utilized.

The exclusion criteria included patients with mental health disorders, a history of spinal surgery, non-idiopathic scoliosis, or scoliosis associated with orthopedic, muscular, neurological, or rheumatic conditions. Radiographs that did not include a complete view of the spine were also excluded.

### 2.3. Morphological and Morphometric Measurements

In addition to collecting demographic data, several key morphological and morphometric parameters were evaluated. These included measurements of the Cobb angle, the determination of the direction of the major spinal curvature, the Lenke classification, and the Risser staging. The Lenke classification system is widely used for assessing adolescent idiopathic scoliosis (AIS) and guiding surgical decisions. It consists of six curve types: type 1 refers to a main thoracic curve, type 2 refers to a double thoracic curve, type 3 refers to a double major curve, type 4 refers to a triple major curve, type 5 refers to a thoracolumbar or lumbar curve, and type 6 refers to a thoracolumbar or lumbar–main thoracic double curve [15].

Each type is further defined by two modifiers: the lumbar spine modifier, which can be A, B, or C, based on the position of the lumbar curve relative to the central sacral vertical line (CSVL), and the sagittal thoracic modifier, which can be −, N, or +, indicating hypokyphosis (less than 10°), normal (10–40°), or hyperkyphosis (greater than 40°) in the thoracic kyphosis angle (T5–T12) [15]. The Risser classification assesses skeletal maturity by evaluating the ossification and fusion stages of the iliac apophysis, divided into six stages, with higher stages indicating greater skeletal maturity. Risser stage 0 indicates no ossification, typically seen in early adolescence, while stage 5 indicates full bony fusion, suggesting skeletal maturity. This classification aids in predicting the risk of curve progression during growth [16].

Measurements of leg length discrepancy, the pelvic axial rotation (PAR) ratio, and the direction of pelvic rotation were performed using the open-source software Horos v. 4.0.0 (https://horosproject.org/). (Access date: 15 January 2025).

**Figure 2:**
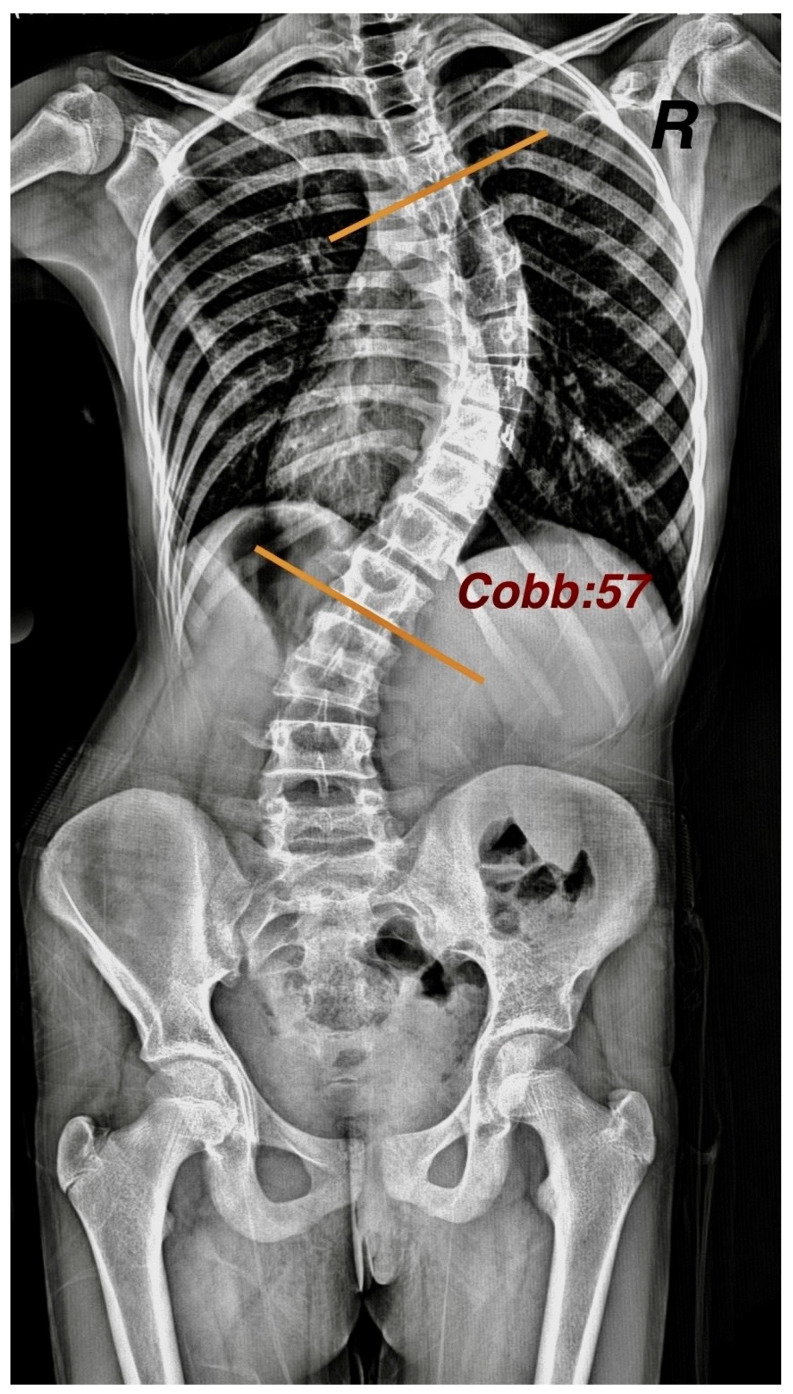
Measurement of Cobb angle on posteroanterior radiograph.

The Cobb angle was calculated in degrees by a specialist orthopedist on a latest spine posteroanterior (PA) radiograph from an archive of individuals in the standing position (Figure 2).

For the Cobb angle measurement, tangents were drawn from the upper limit of the uppermost vertebra included in the curvature and from the lower limit of the lowest vertebra. The angle formed at the intersection of these lines was recorded as the Cobb angle. As scoliosis is clinically defined by a spinal curvature greater than 10°, and no treatment is administered for curves below this threshold in the study clinic, only patients with a Cobb angle exceeding 10° were included.

**Figure 3:**
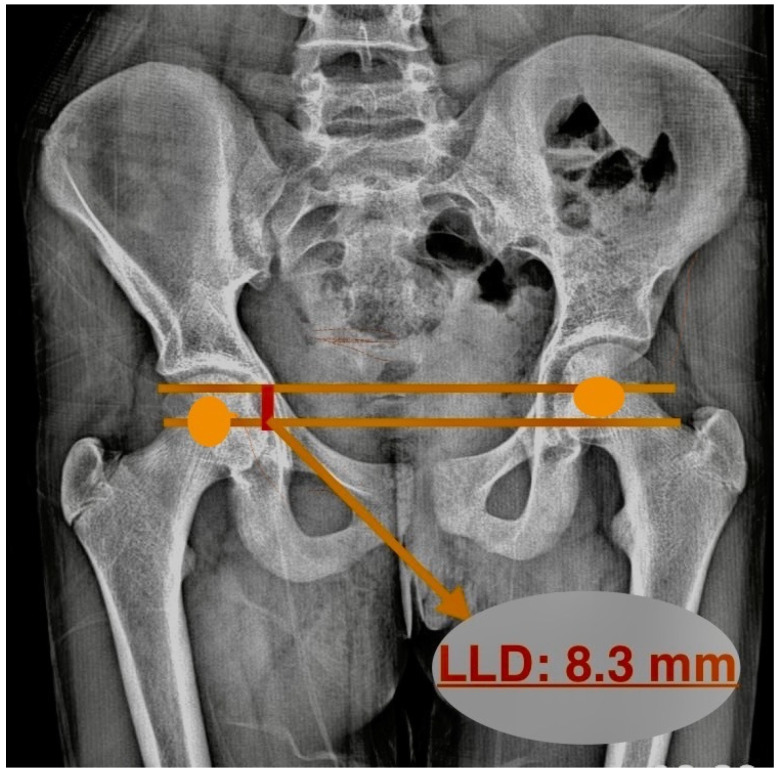
Measuring leg length discrepancy on radiograph.

The LLD was determined by drawing two parallel lines between the centers of the femoral heads. Then, the distance between these lines was measured, and LLD was calculated (Figure 3). Pasha et al. determined this value as the inclination of the bifemoral head axis and substituted it for LLD. While this reflects the apparent limb length, it is susceptible to pelvic tilt and may not represent the true anatomical leg length discrepancy [17].

**Figure 4:**
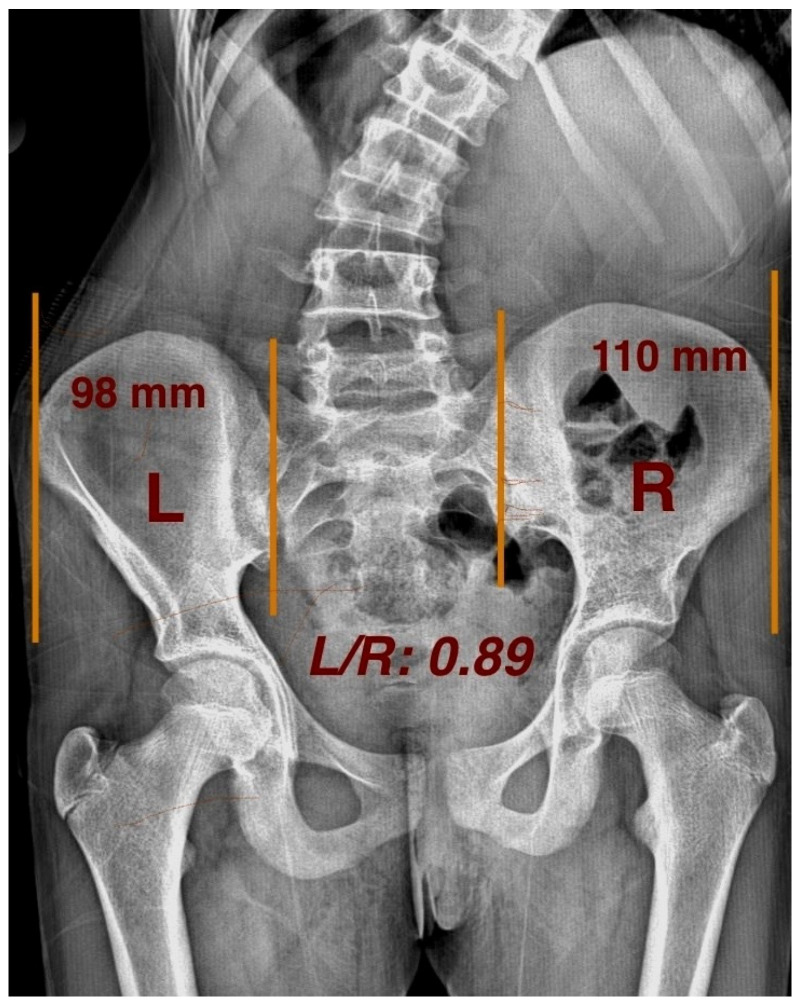
Measuring pelvic axial rotation, and L/R ratio on radiograph.

The pelvic axial rotation (PAR) direction was assessed using the method described by Gum et al. [8]. Studies have verified asymmetrical ilium widths between the convex and concave sides in untreated AIS patients, signifying considerable pelvic rotation [3]. On AP radiographs, the horizontal distance between a vertical reference line drawn from the anterior superior iliac spine (ASIS) and the posterior inferior iliac spine (PIIS) at the corresponding sacroiliac joint was measured to determine the width of the left (L) and right (R) ala ossis ilii (Figure 4).

The PAR direction was then determined by calculating the L/R ratio as follows:

To exclude measurement bias and physiologic PAR, we defined the presence of PAR when the L/R ratio was <0.95 or >1.05.

R-PAR: L/R < 0.95, indicating pelvic rotation toward the right;

L-PAR: L/R > 1.05, indicating pelvic rotation toward the left.

### 2.4. Statistical Analysis

Descriptive statistics were used to summarize the data. Numerical variables are presented as means and standard deviations, while categorical variables are reported as frequencies and percentages. The distribution of the data was assessed using the Shapiro–Wilk test. Since the variables did not follow a normal distribution (*p* > 0.05), non-parametric statistical methods were employed.

The Mann–Whitney U test was used for comparisons between two independent groups, and Chi-square analysis was applied to assess differences between categorical variables. To evaluate the differences among multiple groups, the Kruskal–Wallis test was used, followed by Dunnett T3 post hoc tests to identify specific group differences. Spearman’s correlation analysis was conducted to examine associations between numerical variables. The intraclass correlation coefficient (ICC) was employed to determine the repeatability of measurements.

All statistical analyses were performed using SPSS software (version 25.0, IBM Corp., Armonk, NY, USA). A *p*-value of <0.05 was considered statistically significant.

## 3. Results

Radiographic analysis was conducted on 397 AIS patients, comprising 246 females and 151 males. Pelvic axial rotation (PAR) was directed to the right in 224 patients (88 males, 136 females) and to the left in 173 patients (63 males, 110 females). The intraclass correlations (ICC) were almost perfect for all measurements. The ICC values were LLD (0.82), PAR (0.88) and Cobb angle (0.90). Overall, repeatability was high for all measurements. No statistically significant association was found between the PAR direction and DMC (*p* = 0.919) (Table 1).

Regarding the direction of the major spinal curve, 168 patients exhibited right-sided curves, while 229 had left-sided curves. The major curvature was predominantly left-sided, regardless of whether the PAR was to the right (130 patients) or left (99 patients). Although a significant difference was found between the side with the length discrepancy and the direction of PAR (*p* = 0.001), no significant relationship was observed between the direction of the major curve and PAR direction (*p* = 0.919) (Table 1).

When the Cobb angle and leg length discrepancy were examined according to the PAR direction, no significant differences were found (*p* = 0.737 and *p* = 0.950, respectively). However, when stratified by sex, a significant difference in Cobb angle was found between males and females (*p* = 0.039), while no significant difference was observed in leg discrepancy stratified by sex (*p* = 0.609) (Table 2).

Based on the Lenke classification, the most common curve type was 5C, comprising 135 patients (48 males, 75 females), while types 3B and 3C were the least common, with only two patients each (one male and one female). The evaluation of parameters across Lenke classification groups revealed no significant differences in sex (*p* = 0.991), PAR direction (*p* = 0.673), or leg length discrepancy (*p* = 0.391). (Table 3).

Analysis by Risser classification showed that the highest number of patients were in Risser stage 5 (109 patients), while stage 1 had the fewest (25 patients). There were no significant differences noted between the Risser classification and both the Cobb angle (*p* = 0.733) and leg length discrepancy (*p* = 0.055) (Table 4). Left-sided PAR was slightly more frequent in Risser stage 3, whereas right-sided PAR predominated in all other stages. Nevertheless, no significant relationship was identified between the Risser classification and either PAR direction (*p* = 0.396) or sex (*p* = 0.725) (Table 4).

Assessing the relationship between the parameters, a moderate positive correlation was seen between the Cobb angle and leg length difference (*p* = 0.000, r = 0.600). A very weak negative correlation was observed between the Lenke and age (*p* = 0.005, r = −0.141) and leg length difference and age (*p* = 0.000, r = −0.188). The weakly correlated analyses may exhibit statistical significance; nevertheless, they lack therapeutic relevance (Table 5).

## 4. Discussion

Several studies have discussed how alterations in pelvic positioning can impact spinal balance and alignment [2,18,19]. However, there has been limited research investigating the relationship between PAR and DMC in AIS, with most studies focusing on pre- and post-surgical comparisons [8,10,20]. The current study, which patients with AIS who have not received surgical intervention, offers insights into the morphological characteristics of the spine and pelvis across various degrees of curvature.

The presumed function of pelvic axial rotation (PAR) in three-dimensional spinal balance and compensatory mechanisms has drawn increasing attention in AIS. The direction of thoracic curve rotation and pelvic rotation frequently coincided in seminal works, including those by Gum et al., Wang et al., and Zuckerman et al. [1,8,9]. These studies proposed that the pelvis rotates in the same direction as the main curve, either as a result of a compensatory mechanism or intrinsic pathoanatomic torsion. A significant number of researchers interpret PAR as a reflection of transverse compensation for spinal deformity. An alternative perspective is put forth by Zhao et al. and Asher et al. [10,11].

Contrary to our initial hypothesis, the PAR direction was not significantly associated with Cobb angle, Lenke classification, or DMC, indicating a lack of correlation between pelvic rotation and scoliosis morphology in this cohort. This suggests that in non-operative AIS patients, PAR may be more of a compensatory adaptation than a structural determinant.

Notably, Zuckerman et al. reported an average pelvic rotation of 5.1° in Lenke 1/2 patients, which significantly altered sagittal and rotational spinal parameters but not coronal Cobb angles [9]. However, they also concluded that PAR did not significantly correlate with curve direction, aligning partially with our results but without explicitly contradicting the expected directional association.

Our findings diverge more substantially from the works of Wang et al. and Gum et al., which reported a predominance of right-sided PAR in right thoracic curves (75.8%), a directional consistency between pelvic rotation and main thoracic scoliosis [1,8]. However, when sub-analyzed by Lenke subtypes, even these studies noted inconsistencies. For example, Gum et al. found no significant pelvic rotation in Lenke 1A2 and 2A2 subgroups, suggesting that pelvic rotational compensation may not be universal, especially in curves lacking strong fractional lumbar compensation [8]. In contrast, our study focused on PAR direction as a group for direction determination rather than as a ratio, and numerical variables could not be compared based on the Lenke classification. Nevertheless, no significant difference was noted between the Lenke classification and PAR direction in any group, which might be due to the higher proportion of patients with major thoracic curves in the Lenke classification as per Gum et al.’s study, compared to the higher number of lumbar/thoracolumbar curve patients in our study. In the same study by Gum et al., no correlation was found between the Cobb angle and PAR direction, which aligns with our findings that no such correlation was detected in this study [8].

In our study, among patients with a right-sided major curve, the PAR direction was also on the right in 55.9% of cases, showing a partial alignment with the literature. However, among patients with left-sided major curves, a higher proportion (56.7%) still exhibited right-sided PAR, indicating that the direction of pelvic axial rotation was not consistently aligned with curve direction. Unlike Wang et al., who included only patients with curves ≥40°, our study included patients with curves starting from 10°, which may explain differences in the observed alignment patterns [1]. Research indicates that the direction of PAR is often correlated with the direction of the major scoliotic curve. For example, in patients with a major thoracic curve, the pelvis tends to rotate in the same direction as the apical vertebral rotation [9,17]. This alignment suggests that PAR may be a compensatory mechanism to maintain balance in the coronal and transverse planes and to maintain global balance and prevent further decompensation [17,21]. The magnitude and direction of PAR can influence the progression of scoliotic curves. Studies have shown that patients with asymmetrical PAR are at a higher risk of coronal decompensation after surgical correction, particularly in thoracolumbar and lumbar curves [1,21]. In contrast, for patients with major thoracolumbar or lumbar curves, the relationship between PAR and the major curve is less consistent. Studies have shown that in these cases, the pelvic rotation may not always align with the direction of the major curve, indicating a more complex interplay between spinal and pelvic mechanics [21].

In a study by Asher et al., no significant difference was found between patients with and without increased pelvic rotation in the transverse plane when comparing pre- and post-surgical outcomes [10]. Similarly, the pelvic rotation angle did not significantly impact the efficacy of the surgical intervention, while PAR was altered in particular patients while remaining unchanged in others; however, this did not appear to have a clinical impact [10]. In another study conducted by Qui et al., it was observed that PAR improved slightly in the early postoperative period in patients with AIS and then remained stable [20]. In alignment with our study’s results, Zhao et al. determined that they could not identify a significant association between the primary thoracic curve and PAR, likely attributable to the varying curve types among the patients [11]. The notable positive connection between the proximal thoracic curve and the preoperative PAR is challenging to elucidate. An additional validation of this hypothesis would constitute a compelling occurrence.

A study analyzing axial rotation in 40 individuals with cerebral palsy revealed that the hemipelvis demonstrated greater external rotation in these patients than in the control group, and the severity of neuromuscular scoliosis correlated with PAR [22]. Some studies indicate that PAR deformity must be assessed prior to the acetabular anteversion approach in total hip arthroplasty, emphasizing the importance of meticulous surgical planning, while another study posits that the pelvic incidence may be influenced by pelvic rotation [1,21]. The implementation of a pelvic rotation correction program with Schroth exercises in patients with mild AIS has been shown to enhance deformity repair more efficiently; nevertheless, the morphometric correlation between PAR and scoliosis remains unclear [1,9,21].

The positive correlation between Cobb angle and leg length difference possibly indicated that as the severity of scoliosis increased, asymmetry in the lower extremities also increased. These findings emphasize that pelvic rotation and leg length difference, along with the Cobb angle, should be considered in the assessment of AIS patients. However, our dependence on 2D radiographs and the primitive approach to assessing leg length discrepancy may have added systematic errors attributable to pelvic tilt or rotation, hence constraining the interpretability of correlations with the Cobb angle.

Skeletal asymmetries are commonly observed in AIS patients, with some studies suggesting a relationship between spinal deformities and these asymmetries [8,22]. For example, a study investigating skeletal asymmetry in AIS girls proposed a link between spinal curvature and bone asymmetry [23]. While studies in the literature suggest that LLD exacerbates the Cobb angle, there is no clear evidence demonstrating that AIS induces LLD in advanced curves [24]. Our findings, which show that the leg length discrepancy increases with the Cobb angle, support Burwell et al.’s research on the relationship between leg length differences and scoliosis severity [23]. Further prospective analyses may be needed to clearly understand this situation.

The study’s strengths lie in its large sample size of 397 AIS patients, which enhances the reliability and generalizability of the results. It provides a comprehensive analysis of various scoliosis parameters, including the Cobb angle, Lenke and Risser classifications, LLD, and PAR. Additionally, the use of established classifications and multivariate statistical methods strengthens the validity of the findings.

However, the study’s retrospective design introduces potential biases, such as incomplete data and unaccounted confounders, which limits our ability to establish causal relationships. Its cross-sectional nature also restricts the ability to assess how the relationships between PAR and scoliosis morphometry evolve over time. The findings may have limited generalizability to broader populations, as the study was conducted at a single institution. Furthermore, the study focuses on radiographic parameters without considering other factors like muscle imbalances, postural compensations, or genetic influences. It also does not assess clinical outcomes such as pain, mobility, or quality of life. Moreover, our reliance on 2D radiographs and a basic method for LLD may have introduced systematic errors due to pelvic tilt or rotation, limiting the interpretability of correlations with the Cobb angle. Finally, the study’s limited exploration of the underlying biomechanical mechanisms of pelvic rotation suggests that future research should delve deeper into these aspects for a more complete understanding.

## 5. Conclusions

This retrospective morphometric study contributes to the ongoing discourse on the pathoanatomical intricacies of AIS, specifically interrogating the role of PAR in relation to spinal curve morphology. Contrary to prevailing assumptions, our findings do not support a deterministic or directionally consistent association between PAR and DMC. Our results suggest that PAR should not be regarded as a uniform compensatory mechanism nor as a reliable radiographic surrogate for curve classification or progression. Rather, it appears to reflect a spectrum of biomechanical adaptations that are likely modulated by curve type, skeletal maturity, and possibly by individual variations in neuromuscular control and pelvic morphology. Importantly, the absence of a significant relationship between PAR direction and curve morphology in our cohort does not negate the potential value of pelvic parameters in preoperative assessment, particularly in the context of surgical planning, global alignment restoration, and postoperative coronal decompensation risk stratification. However, our findings underscore the limitations of 2D radiographic analysis in capturing the dynamic and three-dimensional biomechanics of the spine–pelvis–lower limb axis. Future investigations leveraging 3D imaging modalities, gait analysis, and longitudinal follow-up are essential to elucidate the causal and temporal relationships between PAR and curve evolution. A paradigm shift may be warranted—away from viewing PAR as a passive biomechanical epiphenomenon toward considering it a dynamic component of the global compensatory system in AIS. Until such frameworks are validated, spine surgeons should adopt a nuanced, individualized approach when interpreting pelvic morphology in the radiographic assessment of scoliosis patients.

## Figures and Tables

**Table 1:** Distribution of patients’ sex, side with leg length discrepancy, and direction of major curvature according to the direction of pelvic axial rotation.

		PAR Direction			*p*
		Right n (%)	Left n (%)	Total n (%)	
Sex	Male	88 (22.2)	63 (15.9)	151 (38)	0.603
	Female	136 (34.3)	110 (27.7)	246 (62)	
SLLD	Right	174 (43.8)	57 (14.4)	231 (58.2)	<0.001
	Left	48 (12.1)	115 (29.0)	163 (41.1)	
	Symetric	2 (0.5)	1 (0.3)	3 (0.8)	
DMC	Right	94 (23.7)	74 (18.6)	168 (42.3)	0.919
	Left	130 (32.7)	99 (24.9)	229 (57.7)	

PAR: pelvic axial rotation. n: number. SLLD: The side with the leg length discrepancy. DMC: The direction of the major curvature.

**Table 2:** Distribution of Cobb angle and leg length discrepancy in patients according to the direction of pelvic axial rotation and sex.

	PAR Direction	Mean ± SD	*p*	Sex	Mean ± SD	*p*
Cobb angle (°)	Right (n:224)	48.6 ± 15.49	0.737	Male	46.6 ± 13.86	0.039
	Left (n:173)	49.31 ± 15.67		Female	50.32 ± 16.37	
LLD (mm)	Right (n:224)	7.86 ± 7.82	0.950	Male	7.19 ± 6.74	0.609
	Left (n:173)	7.77 ± 7.87		Female	8.21 ± 8.42	

PAR: pelvic axial rotation, SD: standard deviation, LLD: leg length discrepancy.

**Table 3:** Distribution of sex, PAR direction and leg length discrepancy in patients according to the Lenke classification.

Lenke Classification	Sex			PAR Direction			LLD	
	Male n (%)	Female n (%)	*p*	Right n (%)	Left n (%)	*p*	Mean ± SD	*p*
1A	11 (2.8)	22 (5.5)	0.991	21 (5.3)	12 (3)	0.673	6.78 ± 7.58	0.391
1B	16 (4)	18 (4.5)		20 (5)	14 (3.5)		6.80 ± 6.46	
1C	12 (3.0)	19 (4.8)		17 (4.3)	14 (3.5)		5.22 ± 5.31	
2A	3 (0.8)	5 (1.3)		5 (1.3)	3 (0.8)		8.20 ± 13.40	
2B	10 (2.5)	16 (4)		14 (3.5)	12 (3)		9.07 ± 6.38	
2C	19 (4.8)	33 (8.3)		22 (5.5)	30 (7.6)		8.26 ± 6.92	
3A	5 (1.3)	6 (1.5)		5 (1.3)	6 (1.5)		9.11 ± 8.41	
3B	1 (0.3)	1 (0.3)		2 (0.5)	0 (0)		1.09 ± 0.52	
3C	1 (0.3)	1 (0.3)		2 (0.5)	0 (0)		11.1 ± 11.24	
4A	4 (1)	4 (1)		4 (1)	4 (1)		7.54 ± 7.40	
4B	5 (1.3)	10 (2.5)		8 (2)	7 (1.8)		7.11 ± 5.24	
4C	4 (1)	11 (2.8)		10 (2.5)	5 (1.3)		8.64 ± 11.42	
5C	48 (12.1)	75 (18.9)		71 (17.9)	52 (13.1)		7.88 ± 8.51	
6C	12 (3)	25 (6.3)		23 (5.8)	14 (3.5)		9.92 ± 8.47	
Total	151 (38)	246 (62)		224 (56.4)	173 (43.6)			

PAR: pelvic axial rotation, LLD: Leg length discrepancy, SD: standard deviation, n: number.

**Table 4:** Distribution of sex, PAR direction, Cobb angle, and leg length discrepancy in patients according to the Risser classification.

Risser Classification	Sex			PAR Direction			Cobb Angle		LLLD	
	Male n (%)	Female n (%)	*p*	Right n (%)	Left n (%)	*p*	Mean ± SD	*p*	Mean ± SD	*p*
1	11 (2.8)	14 (3.5)	0.725	16 (4)	9 (2.3)	0.396	50.79 ± 16.37	0.733	6.94 ± 8.99	0.055
2	27 (6.8)	46 (11.6)		46 (11.6)	27 (6.8)		48.91 ± 12.1		7.63 ± 6.36	
3	33 (8.3)	42 (10.6)		36 (9.1)	39 (9.8)		49.85 ± 15.29		7.90 ± 6.66	
4	41 (10.3)	74 (18.6)		64 (16.1)	51 (12.8)		48.93 ± 16.82		9.09 ± 8.65	
5	39 (9.8)	70 (17.6)		62 (15.6)	47 (11.8)		47.79 ± 16.39		6.76 ± 8.20	
Total	151 (38)	246 (62)		224 (56.4)	173 (43.6)					

Same superscript letters show values with statistically significant differences per property. PAR: Pelvic axial rotation. LLD: Leg length discrepancy. SD: Standard deviation. n: Number.

**Table 5:** Correlation of parameters with each other.

		PAR Direction	SLLD	LLD	Lenke Classification	Risser Classification	Age	DMC	Sex
Cobb angle	r	0.017	−0.069	0.600	0.098	−0.069	−0.502	0.054	0.104
	*p*	0.738	0.170	0.000	0.052	0.171	0.064	0.285	0.039
PAR direction	r		0.444	0.003	−0.018	0.026	0.019	−0.008	0.029
	*p*		0.000	0.951	0.724	0.605	0.707	0.872	0.560
SLLD	r			−0.047	−0.062	0.006	0.015	−0.047	−0.025
	*p*			0.349	0.219	0.905	0.767	0.346	0.622
LLD	r				0.068	−0.071	−0.078	0.064	0.026
	*p*				0.176	0.160	0.121	0.205	0.610
Lenke classification	r					−0.141	−0.141	0.751	0.023
	*p*					0.005	0.005	0.000	0.654
Risser classification	r						0.902	−0.105	0.042
	*p*						0.000	0.037	0.406
Age	r							−0.109	−0.266
	*p*							0.030	0.000
DMC	r								0.033
	*p*								0.518

PAR: pelvic axial rotation, SLLD: The side with the leg length discrepancy, LLD: Leg length discrepancy, DMC: The direction of the major curvature.

## Data Availability

The datasets used and/or analyzed during the current study are available from the corresponding author on reasonable request.

## Abbreviations

The following abbreviations are used in this manuscript:

AIS	Adolescent idiopathic scoliosis
PAR	Pelvic axial rotation
ASIS	Anterior superior iliac spine
PSIS	Posterior inferior iliac spine
LLD	Leg length discrepancy
SLLD	The side with the leg length discrepancy
DMC	The direction of the major curvature
